# Effect of transition metal alloying elements on the deformation of Ti-44Al-8Nb-0.2B-0.2Y alloys

**DOI:** 10.1038/s41598-018-32570-4

**Published:** 2018-09-24

**Authors:** Laiqi Zhang, Gengwu Ge, Junpin Lin, Mark Aindow, Lichun Zhang

**Affiliations:** 1 State Key Laboratory for Advanced Metals and Materials, University of Science and Technology Beijing, Beijing, 100083 China; 2 Department of Materials Science and Engineering, Institute of Materials Science, University of Connecticut, Storrs, CT 06269-3136 USA

## Abstract

A series of β-γ Ti-44Al-8Nb-0.2B-0.2Y alloys have been modified with 0.5 to 2.0 at.% of the β-stabilizing elements Mn, Cr, Mo and V. Additions of Cr and Mo alone result in a decrease in the flow stress, whereas the opposite effect was observed for additions of V. For alloys with Mn additions, a minimum value of the flow stress was achieved in the alloy with 1.5% Mn. For alloys with combined additions, optimum hot deformation behavior was obtained for the alloy with 1.5% Mn and 1.0% Cr.

## Introduction

TiAl-based alloys exhibit excellent elevated temperature strengths making them promising candidates for high-temperature structural applications in the aerospace and automotive industries^1,2^. However, their inherent brittleness and poor hot deformability limit their use^3,4^. Accordingly, many different approaches, such as alloying^5,6^, heat treatment^7^ and thermo-mechanical treatment^8^, have been investigated in an attempt to produce fine, homogeneous microstructures with better properties. More recently, there has been an increased emphasis on the development of β-γ TiAl alloys, which exhibit good hot workability and fine as-cast microstructures through β solidification^9–14^. It was reported that the addition of β-stabilizing elements such as Nb, Mn, Cr, Mo and V led to an enlargement of the β phase region, as well as partial stabilization of the β phase to room temperature, which modifies the microstructure significantly^9,10^. Due to the number of independent slip systems, the disordered bcc β phases are expected to be much softer than α and γ phases at elevated temperature, and should thus contribute to the enhancement of hot deformability. Indeed, such β-modified alloys exhibited greatly improved room temperature ductility and hot deformability^15^, as compared with conventional duplex α_2_-γ TiAl alloys, despite the fact that the β phase is usually present as an ordered B2 phase at room temperature.

Previous work has shown that Ti-Al alloys containing high Nb contents exhibit better strength and oxidation resistance at temperatures up to 1000 °C than conventional TiAl alloys^16^. There have been some studies on the effect of hot deformation on the microstructure of such β-γ TiAl alloys^17–21^, however, there have been few studies on the effect of β stabilizing elements, such as Mn, Cr, Mo and V, on the hot deformability of β-γ TiAl alloys with high Nb contents.

## Results and Discussion

**Figure 1:**
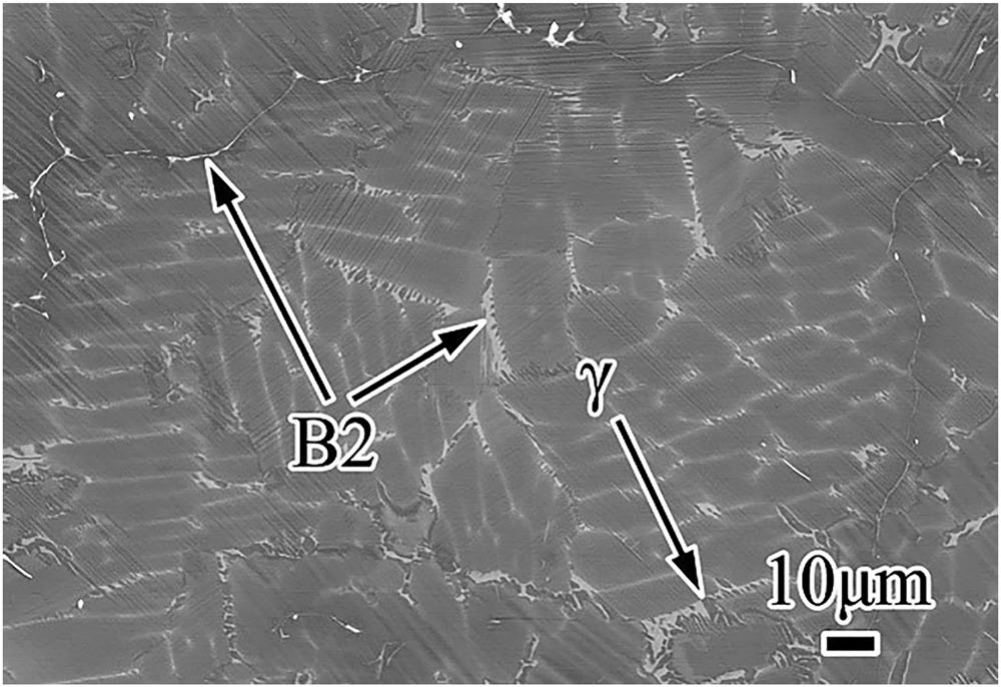
BSE SEM micrograph of the microstructure in the as-cast Ti-44Al-8Nb-0.2B-0.2Y base alloy.

The as-cast base alloy (#1: Ti-44Al-8Nb-0.2B-0.2Y) exhibits equiaxed grains with dominant fine lamellar colonies and mixtures of ordered B2 (white) and γ (gray) phases (Fig. 1), which were identified individually by EDXS analysis. The fine lamellar colonies arise due to the twelve different orientation variants of α formed during the β → α transformation^11^, while the β phase refinement by B restricts the growth of the α lamellae after the transformation. Meanwhile, the mixture of B2 and γ grains are formed by a discontinuous β/B2 → γ reaction during solidification. The high Nb content favors alloy solidification through the β phase pathway, so the β phases are dispersed inside the lamellar colonies, as well as at the boundaries of lamellar colonies and at the triple junctions. These β grains at the boundaries arise due to incomplete transformation of primary β → α during rapid cooling, whereas the β grains within the lamellar colonies precipitate from α laths during the α → β +  γ decomposition^22^.

To illustrate the effects of alloying additions we consider firstly alloys #2, #3, #4 and #5, which have 0.5, 1.0, 1.5 and 2.0 at.% Mn, respectively. The microstructures of the as-cast and deformed samples for these alloys are shown in (Fig. S1a–h), respectively. For the as-cast alloys #2, #3 and #5 the β phases exhibit the lath-like or acicular morphologies, and are mainly dispersed inside the lamellar colonies, as shown in (Fig. S1a,c,g) respectively. However, some coarse β phases are also distributed in the microstructure of alloy #5. In contrast, alloy #4 with 1.5 at.% Mn contains network β phase distributed primarily at the boundaries of the lamellar colonies and at the triple junction areas, as shown in Fig. S1e. From the corresponding deformed microstructures, dynamic recrystallization (DRX) has apparently occurred during high-temperature deformation; the amount of β phase after deformation is significantly higher and we ascribe this to recrystallization processes breaking down the lamellar colonies into fine γ and β phases. Meanwhile, the deformed microstructure exhibits bending, elongation, kinking and coarsening of the β phase, indicating that this phase is softer than the α_2_ and γ phase. This is expected since the bcc β phase exhibits sufficient independent slip systems to accommodate any arbitrary deformation. Correspondingly, preferential deformation of the soft β phase prior to the α_2_ phase and γ phase during the hot deformation process can inhibit the formation of stress concentrations and prevent the failure of lamellar colonies by cracking.

**Figure 2:**
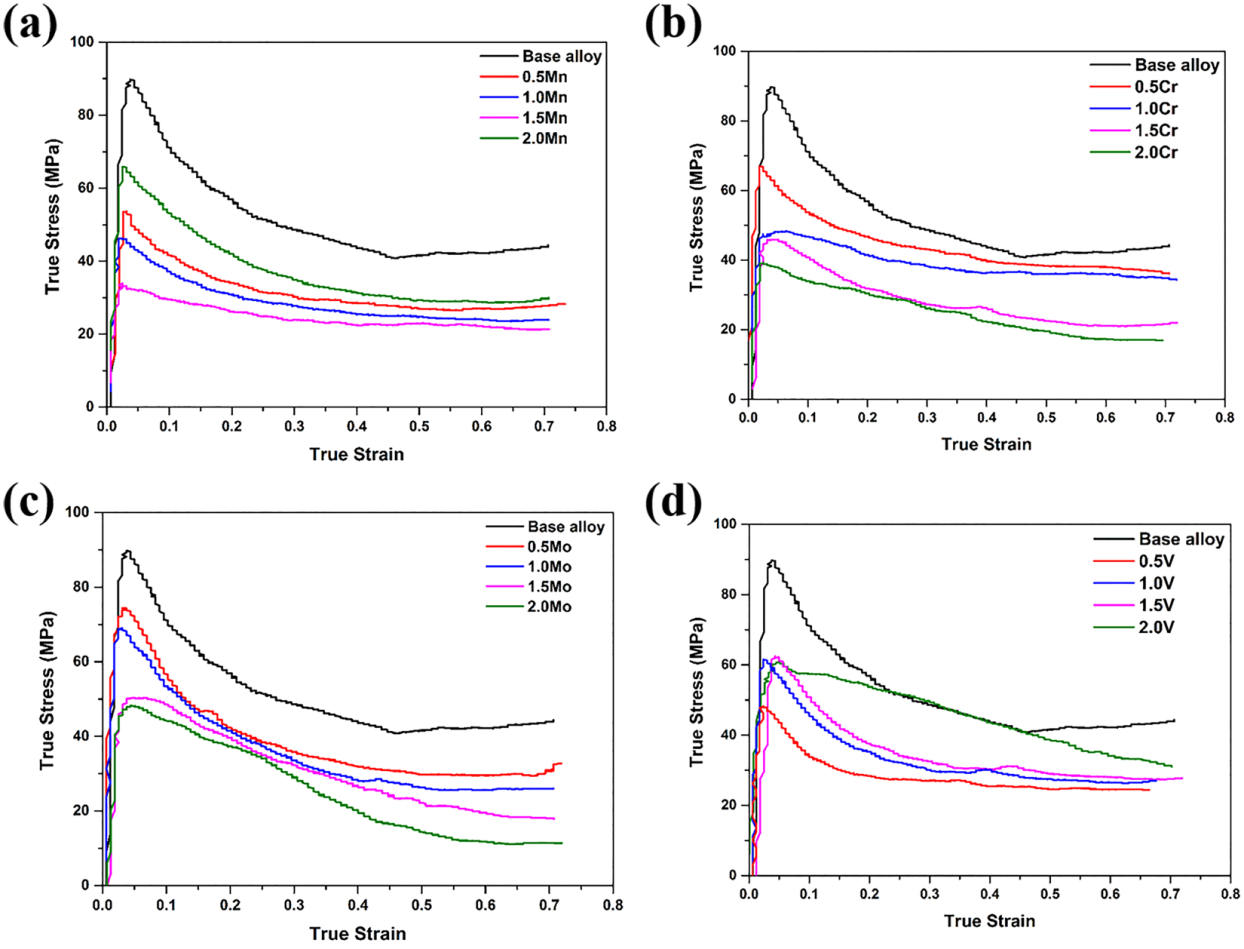
True stress-strain curves obtained at $\dot{\varepsilon }$ = 0.001 s^−1^ from alloys containing 0.5–2.0% of: (**a**) Mn, (**b**) Cr, (**c**) Mo, (**b**) V.

The true stress – true strain curves obtained from alloys #1–5 are shown in Fig. 2(a). In each case, there are three stages to the deformation: first the flow stress increases continuously with strain to a peak stress value, then decreases gradually, and eventually reaches a steady state. These curves exhibit typical work hardening and flow softening features, but the effect of the Mn content is remarkable. Initially, the peak stress decreases with increasing Mn content, with a minimum peak stress for alloy #4 (1.5 at.% Mn), but then increases dramatically for alloy #5. The peak stress is a measure of the hot deformability of an alloy, with lower values being more favorable. The initial decrease in peak stress with increasing Mn content could be explained on the basis of the increase in the volume fraction of the softer β phase. However, we would expect the highest β phase volume fraction for alloy #5, which has a significantly higher peak stress. Thus, it is not only the amount of the β phase that determines the hot deformation characteristics, but also the way in which this phase is distributed.

Equivalent BSE SEM micrographs and true stress – true strain curves from the alloys with Cr additions (alloys #6–9) are shown in Figs S2 and 2(b), respectively. The as-cast microstructures contain white laths of β phase dispersed finely in the matrix, and the volume fraction of these features increases with Cr content. For the alloys with 0.5 and 1.0 at.% Cr (#6 and #7, respectively), the deformed microstructure contains both remnant lamellae and new recrystallized grains, indicating that the microstructure has undergone partial DRX. For alloys containing 1.5 and 2.0% Cr, (#8 and #9, respectively), the microstructure has undergone more complete DRX, and is dominated by grains of γ and β/B2 phases, with almost no residual α phase. In this case, the increase in the volume fraction of the β phase with increasing Cr content leads to a corresponding continuous decrease in the peak stress values for these alloys (i.e. the alloy with 2.0 at.% Cr has the best hot deformability).

The BSE SEM micrographs from the alloys with Mo additions (alloys #10–13) are shown in Fig. S3. There are no obvious changes in the volume fraction and the morphology of the β phase in the as-cast microstructures of these alloys, and the deformed microstructures show only partial recrystallization with some lamellar colonies being preserved. The corresponding true stress-strain curves in Fig. 2(c) show a corresponding continuous decrease in the peak stress values for these alloys with increasing Mo content, but the magnitude of this effect is less than for Cr additions.

Rather different effects are observed for the alloys with V additions (alloys #14–17). In the BSE SEM micrographs from the as-cast microstructures, for V contents of 0.5 and 1.0% (alloys #14 and #15), the fine lath β phase is mainly dispersed along the lamellar colonies boundaries, as well as in a few β phase grains at the triple junctions (Fig. S4). However, for V contents of 1.5 and 2.0% (alloys #16 and #17), coarse β phase grains are found, and these are not distributed evenly in the matrix. These microstructural effects may help to account for the deformation characteristics of the alloys. As shown in Fig. 2(d), the lowest peak stress is recorded for the alloy with 0.5% V, and the peak values for the other three alloys are approximately equal.

**Figure 3:**
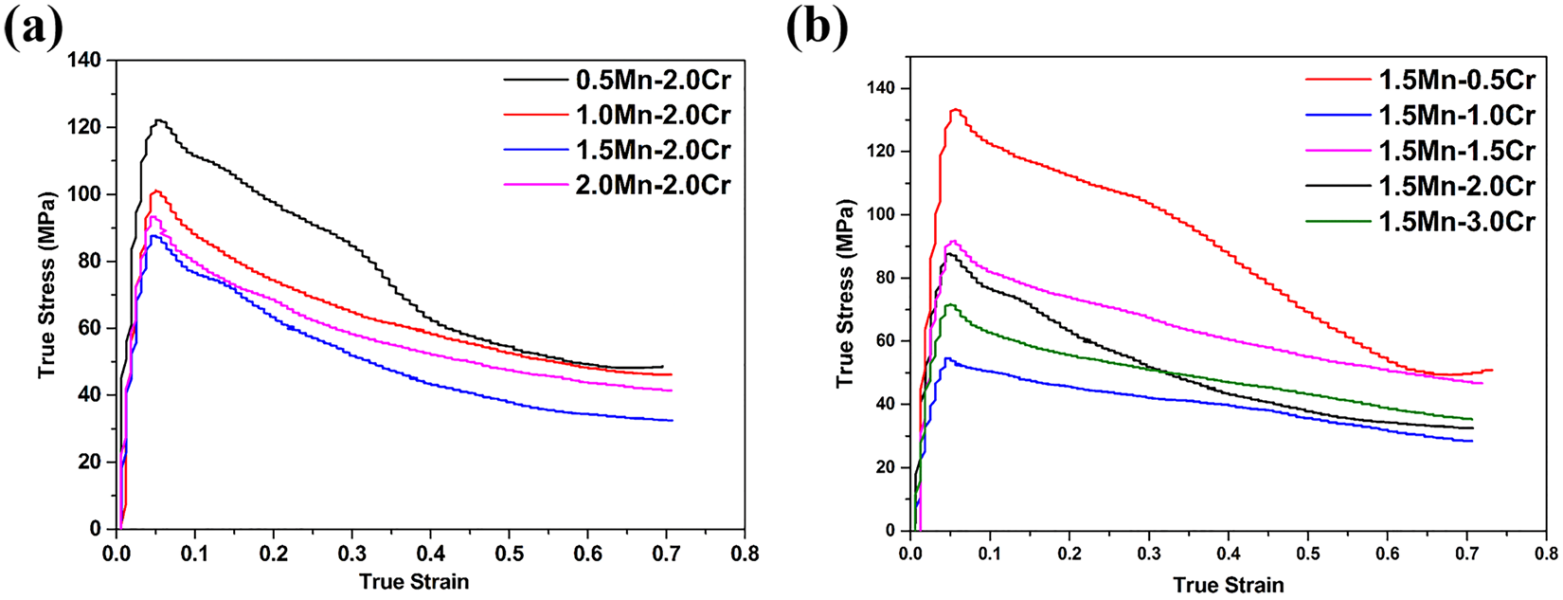
True stress-strain curves obtained at $\dot{\varepsilon }$ = 0.05 s^−1^ from alloys containing: (**a**) 2.0 Cr plus 0.5–2.0%Mn, (**b**) 1.5% Mn plus 0.5–3.0% Cr.

Since additions of Mn and Cr improve the hot deformability more significantly than Mo or V, we next investigated possible combined effects for alloys with both Mn and Cr additions. The lowest flow stress values obtained with single additions were for alloys with 1.5% Mn and 2.0% Cr. As such, alloys were produced with 2.0% Cr plus 0.5–2.0% Mn (alloys #18–21), and with 1.5% Mn plus 0.5–3.0% Cr (alloys #22–25). The BSE SEM images from alloys #18–21 (Fig. S5) show that for low Mn contents the β phase is finely dispersed, but the volume fraction is low. Conversely for Mn contents of 1.5 and 2.0%, the volume of β is greater and the phase exhibits a dendritic or network morphology. These latter alloys exhibit similar moderately decreased peak stresses (Fig. 3(a)). The BSE SEM images from alloys #22–25 (Fig. S6) show that the volume fraction of the β phase increases with Cr content, but the β phase is fine and distributed uniformly when the Cr content is 0.5 or 1.0%, whereas it is coarser and exhibits a dendritic morphology with the Cr content is 1.5 or 2.0%. These differences are reflected in the true stress – true strain curves (Fig. 3(b)), which show a complex variation in the peak stress with Cr content. What does emerge is that the lowest overall value of the peak stress is for alloy #23, which contains 1.5% Mn and 1.0% Cr. All of the deformed microstructures for alloys with both Mn and Cr exhibit at least partial DRX (Figs S5 and S6), but the volume fraction and morphology of the β phase varies in a more complex fashion than for alloys with single additions of β phase stabilizers. Thus alloy #23, which exhibits the lowest overall value of the peak stress, does not have the highest volume fraction of the β phase. Instead, it seems to exhibit an intermediate β phase volume fraction, but this phase is divided finely and distributed uniformly throughout the microstructure.

**Figure 4:**
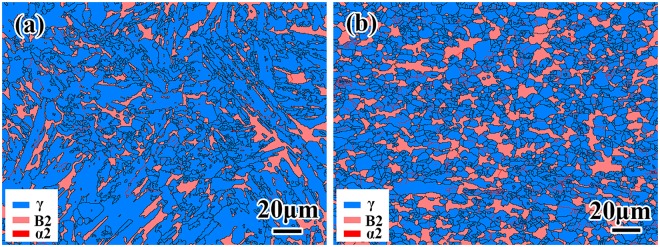
EBSD phase maps for the optimized Ti-44Al-8Nb-1.5Mn-1.0Cr-0.2B-0.2Y alloy in the: (**a**) as-cast, and (**b**) deformed conditions.

**Figure 5:**
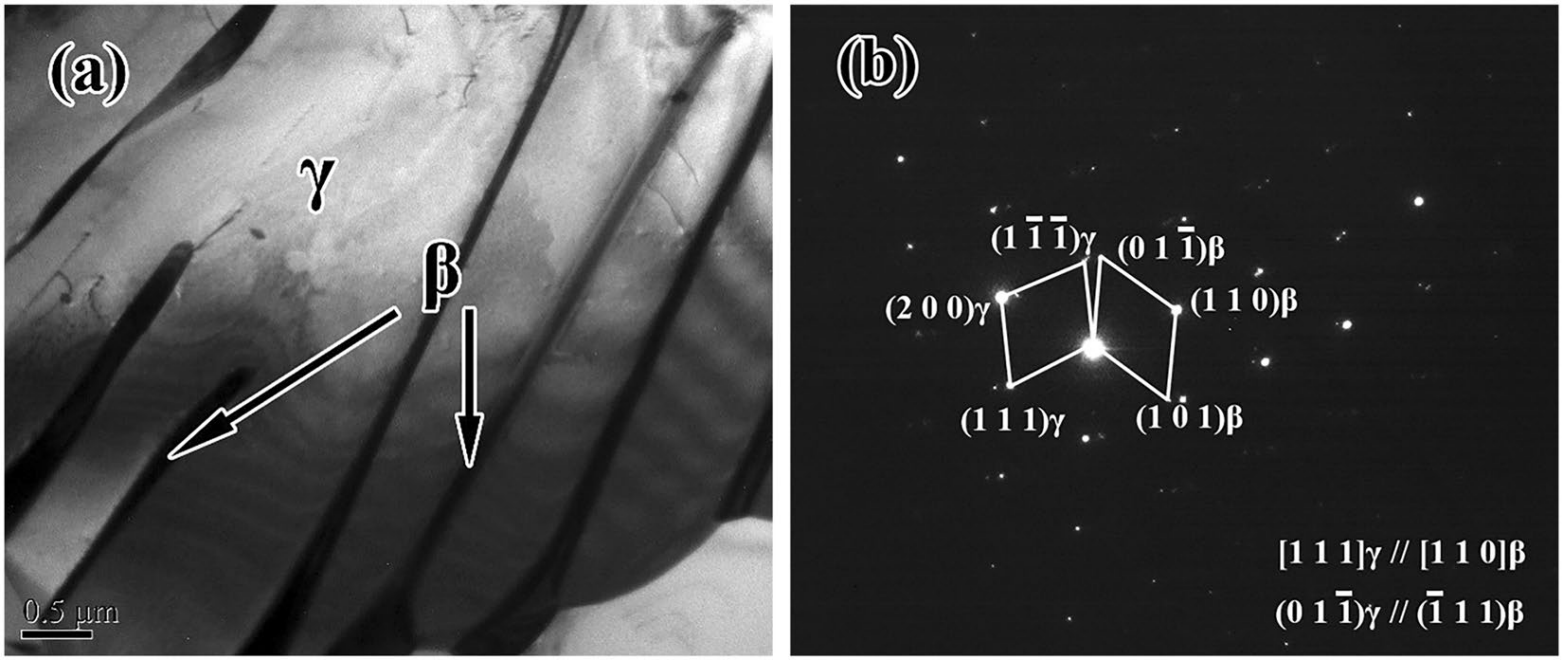
Microstructure at the center of the button for the optimized alloy: (**a**) bright field TEM image (**b**) SADPs of the β/B2 and γ phases.

In the remainder of this report, we concentrate upon the characteristics of this one “optimized” alloy. A combination of SEM/EBSD and TEM imaging/diffraction techniques was used to evaluate the microstructures exhibited by this alloy in more detail. Phase and boundary fractions for the optimized alloy in the as-cast and deformed conditions are shown in Table 1. An example of an EBSD phase map obtained from an electropolished sample of the as-cast ingot is presented in Fig. 4(a). Such maps confirm that the microstructure of the ingot is mainly composed of the dominant γ (85.0 vol.%) and β/B2 (14.9 vol.%) phases. Clearly, the β phase is located at the boundaries of the lamellar γ colonies and within these colonies. There is almost no α phase detected by EBSD. The distribution of the β/B2 phase was further confirmed by TEM on electro-polished foils of the as-cast microstructure, and examples of these data are shown in Fig. 5. Figure 5(a) is a bright field image from the center of the button, and Fig. 5(b) is an example of a selected area diffraction pattern from a regions containing the β and γ phases. Such images show that the β phase is present as a layer approximately 0.2 µm in thickness at the boundaries of the γ phase laths. The relative orientation between adjacent β phase and γ phase corresponds to a relationship where (111)*γ*//(110)β.

**Table 1:** Phases and boundaries fractions for the optimized Ti-44Al-8Nb-1.5Mn-1.0Cr-0.2B-0.2Y alloy in the as-cast and deformed conditions.

	Phases Fraction			Boundaries Fraction	
	γ Phase	β Phase	α Phase	HAGBs	LAGBs
As-cast	0.850	0.149	0.001	0.944	0.056
As-deformed	0.760	0.239	0.001	0.847	0.153

The corresponding EBSD map for the deformed alloy is shown in Fig. 4(b): the volume fraction of the γ phase is lower (76.0%) and that of the β/B2 is correspondingly higher (23.9%). The other main change is in the distribution of grain boundary types. There is a significant increase in the proportion of low-angle grain boundaries (LAGBs, marked by red lines) with misorientations of 2–15° from 5.6% to 15.3%. While this may appear to be inconsistent with the DRX process invoked previously, we note that the number of high angle grain boundaries (HAGBs, marked by black lines) with misorientations of >15° also increases. Thus the DRX process involves a balance between dislocation generation (to accommodate the deformation), dislocation rearrangement (to form LAGBs), and nucleation of new defect-free grains defined by HAGBs (i.e. recrystallization). The net effect of these processes is to break down the lamellar colonies into fine γ and B2 grains during hot deformation as observed experimentally.

## Conclusions

In conclusion, β-γ TiAl alloys are known to exhibit far superior hot deformability to conventional γ TiAl alloys. Here we have considered the effects of adding Mn, Cr, Mo and V as β stabilizing elements to a Ti-44Al-8Nb-0.2B-0.2Y alloy (all in at.%). The as-cast and as-deformed microstructures, as well as the hot deformation behaviors of the alloys were investigated systematically. The true stress – true strain curves obtained from each of the alloys exhibited typical work hardening and flow softening features. For alloys with Mn additions only, the minimum peak stress value was obtained at 1.5% Mn, which can be ascribed to the combined effect of the amount and morphology of the β phase. For alloys with additions of Cr or Mo alone the peak flow stress decreases with increasing alloy content, however for alloys with V additions the opposite effect was observed. The additions of Mn and Cr gave the lowest peak yield stress corresponding to the best hot workability. For alloys with both Mn and Cr additions, the combination of 1.5% Mn and 1.0% Cr gave the lowest peak flow stress due to the β phase being finer and distributed more uniformly than in any of the other alloys considered. These results demonstrate how small modifications in alloy composition can be used to control the β phase volume fraction and morphology in β-γ TiAl alloys and to optimize the hot deformability of these materials.

## Materials and Methods

Here we consider the microstructure and deformation behavior of a series of 25 Ti-44Al-8Nb-0.2B-0.2Y alloys (all in at.%) modified with 0–2.0% of the β stabilizing elements: Mn, Cr, Mo and/or V. The nominal compositions of the alloys are presented in Table 2. The as-cast alloys were prepared as buttons in a non-consumable arc vacuum melting furnace under an argon atmosphere. The buttons were melted five times to ensure chemical homogeneity. Cylindrical compression specimens 6 mm in diameter and 9 mm long were cut from the buttons by electrical discharge machining and the surfaces were then ground and polished to a mirror finish. Compression tests were conducted in a Gleeble 1500 machine at a temperature of 1200 °C and a strain rate of $\dot{\varepsilon }$ = 0.001 s^−1^ to an engineering strain of 50%. Samples with combinations of β stabilizing elements, which exhibited better hot deformability, were tested at $\dot{\varepsilon }$ = 0.05 s^−1^, with all other conditions unchanged. The compressed specimens were water quenched immediately after the tests to preserve the deformed microstructures.

**Table 2:** The nominal composition for the alloys considered in this study (all in at. %), together with the measured volume fraction (V_f_) of the β phase.

Alloy	Ti	Al	Nb	Mn	Cr	Mo	V	B	Y	V_f_ β (%)
1	47.6	44	8.0	0	0	0	0	0.2	0.2	3.2
2	47.1	44	8.0	0.5	0	0	0	0.2	0.2	4.7
3	46.6	44	8.0	1.0	0	0	0	0.2	0.2	10.0
4	46.1	44	8.0	1.5	0	0	0	0.2	0.2	12.5
5	45.6	44	8.0	2.0	0	0	0	0.2	0.2	17.6
6	47.1	44	8.0	0	0.5	0	0	0.2	0.2	11.0
7	46.6	44	8.0	0	1.0	0	0	0.2	0.2	13.5
8	46.1	44	8.0	0	1.5	0	0	0.2	0.2	14.2
9	45.6	44	8.0	0	2.0	0	0	0.2	0.2	17.9
10	47.1	44	8.0	0	0	0.5	0	0.2	0.2	12.5
11	46.6	44	8.0	0	0	1.0	0	0.2	0.2	13.6
12	46.1	44	8.0	0	0	1.5	0	0.2	0.2	15.2
13	45.6	44	8.0	0	0	2.0	0	0.2	0.2	16.3
14	47.1	44	8.0	0	0	0	0.5	0.2	0.2	8.5
15	46.6	44	8.0	0	0	0	1.0	0.2	0.2	10.2
16	46.1	44	8.0	0	0	0	1.5	0.2	0.2	16.3
17	45.6	44	8.0	0	0	0	2.0	0.2	0.2	18.1
18	45.1	44	8.0	0.5	2.0	0	0	0.2	0.2	11.3
19	44.6	44	8.0	1.0	2.0	0	0	0.2	0.2	11.9
20	44.1	44	8.0	1.5	2.0	0	0	0.2	0.2	23.0
21	43.6	44	8.0	2.0	2.0	0	0	0.2	0.2	28.3
22	45.4	44	8.0	1.5	0.5	0	0	0.2	0.2	12.3
23	45.1	44	8.0	1.5	1.0	0	0	0.2	0.2	13.2
24	44.6	44	8.0	1.5	1.5	0	0	0.2	0.2	21.6
25	43.1	44	8.0	1.5	3.0	0	0	0.2	0.2	26.2

The microstructures of the as-cast and the deformed specimens were characterized by back-scattered electron (BSE) imaging in a Zeiss Supra 55 scanning electron microscope (SEM) operating at an accelerating voltage of 30 kV and equipped with an Oxford X-Max energy dispersive X-ray spectroscopy (EDXS) system and a HKL fast acquisition electron backscattered diffraction (EBSD) system. Samples for SEM were prepared by conventional metallographic grinding and polishing, whereas samples for EBSD were electro-polished using an electrolyte of 65 vol.% methanol, 30 vol.% butanol, and 5 vol.% perchloric acid at a temperature of between −20 and −30 °C, and a potential of 30 V. The EBSD data were collected at intervals of 0.5 μm and were analyzed using HKL Channel5 software. Samples for transmission electron microscopy were prepared by mechanical polishing and then twin-jet electro-polishing to perforation the same electrolyte and condition as for the EBSD samples. TEM experiments were performed on a Tecnai G2 F30 field emission transmission electron microscope operating at an accelerating voltage of 300 kV. The volume fraction of the β phase was evaluated using the image processing software Image J.

## Electronic supplementary material

Supplementary Information
