# Supplementary material for: Effect of transition metal alloying elements on the deformation of Ti-44Al-8Nb-0.2B-0.2Y alloys
Source: Sci Rep. 2018 Sep 24;8:14242. doi: 10.1038/s41598-018-32570-4 (PMC6155326; doi:10.1038/s41598-018-32570-4)
Supplement: Supplementary file 1 — Supplementary Information [file 41598_2018_32570_MOESM1_ESM.pdf]

Supplementary materials

Effect of transition metal alloying elements on the deformation of Ti-44Al-8Nb-0.2B-0.2Y alloys

Laiqi Zhang[1], Gengwu Ge[1], Junpin Lin[1], Mark Aindow[2], Lichun Zhang[2]

[1]*State Key Laboratory for Advanced Metals and Materials, University of Science and Technology Beijing, Beijing 100083, China*

[2]*Department of Materials Science and Engineering, Institute of Materials Science, University of Connecticut, Storrs, CT 06269-3136, USA*

*Corresponding author: Prof. Laiqi Zhang

TEL: +86 10 62334925; FAX: +86 10 62333447

E-mail: zhanglq@ustb.edu.cn

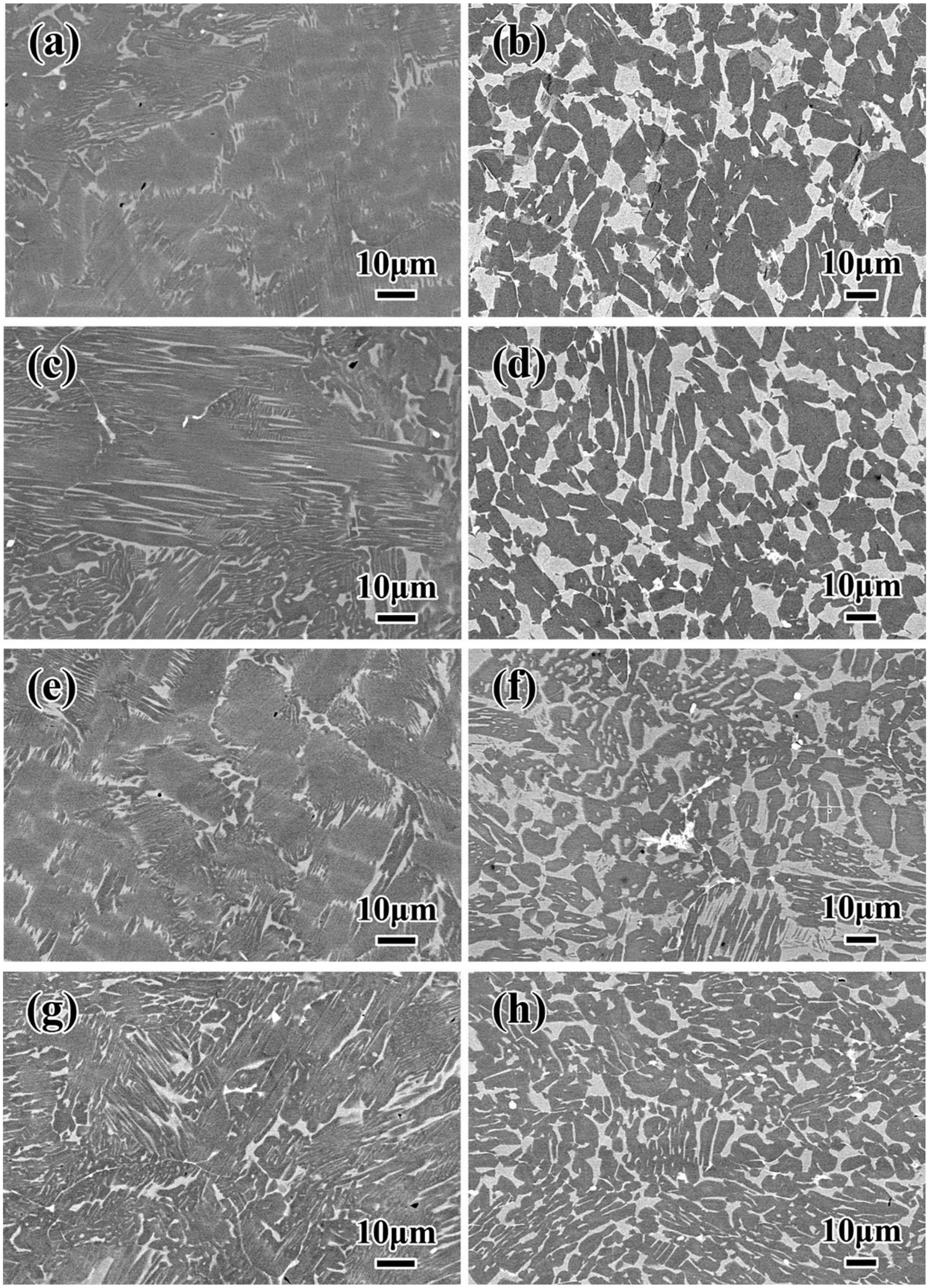

Supplementary Figure S1. BSE SEM images of the as-cast (a,c,e,g) and as-deformed (b,d,f,h) microstructures for alloys with Mn additions of: (a,b) 0.5%, (c,d) 1.0%, (e,f) 1.5%, (g,h) 2.0%.

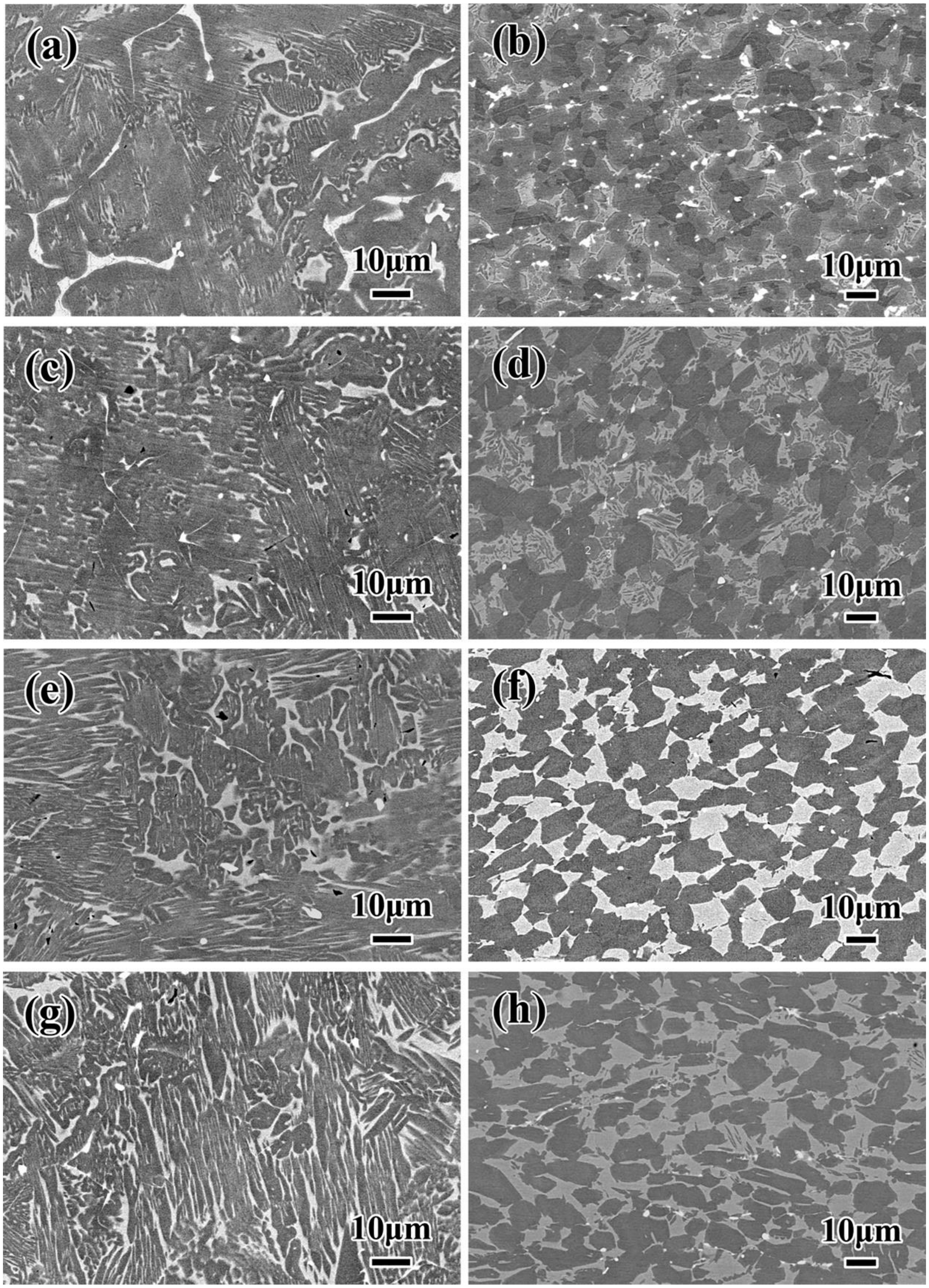

Supplementary Figure S2. BSE SEM images of the as-cast (a,c,e,g) and as-deformed (b,d,f,h) microstructures for alloys with Cr additions of: (a,b) 0.5%, (c,d) 1.0%, (e,f) 1.5%, (g,h) 2.0%.

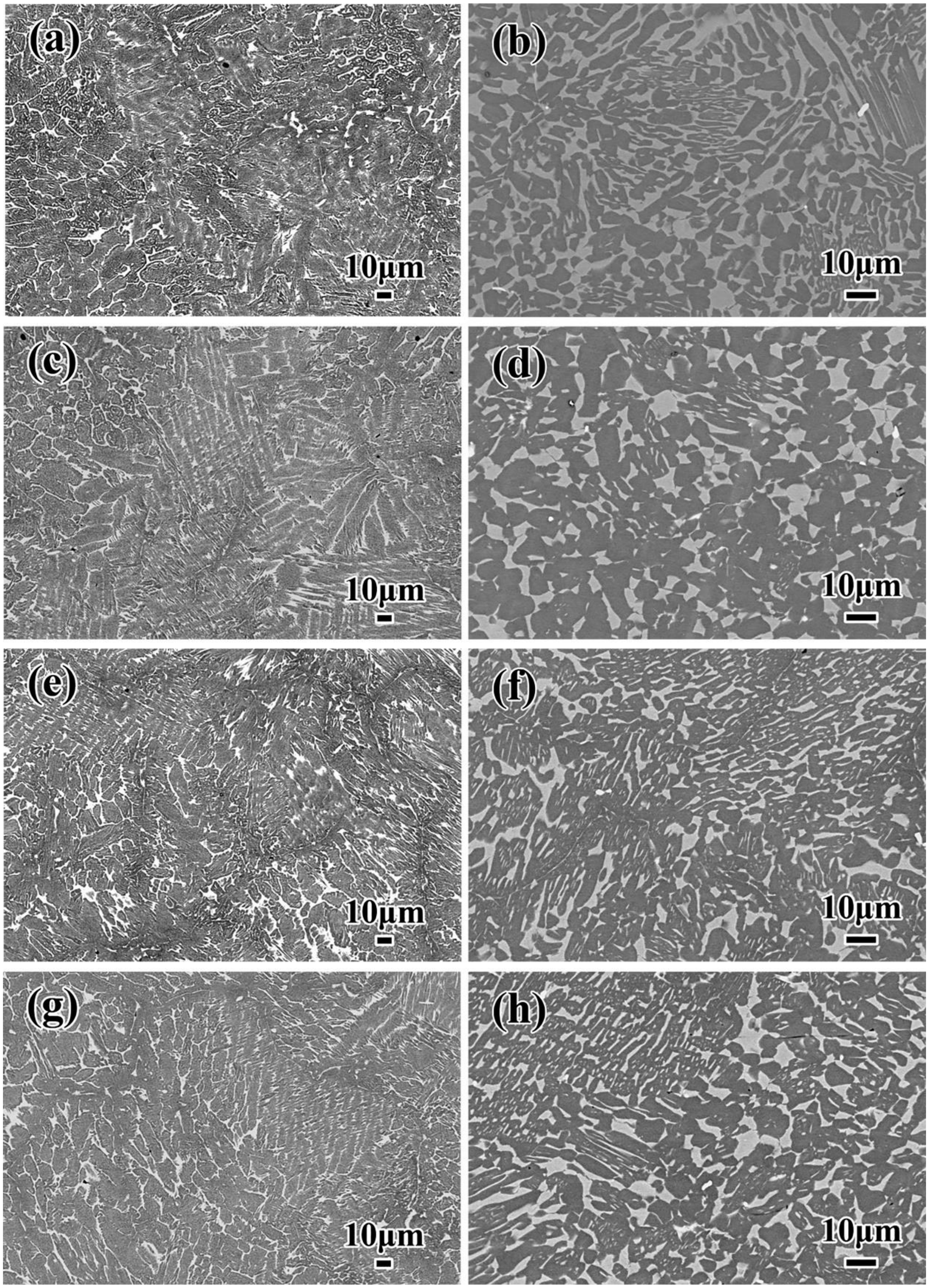

Supplementary Figure S3. BSE SEM images of the as-cast (a,c,e,g) and as-deformed (b,d,f,h) microstructures for alloys with Mo additions of: (a,b) 0.5%, (c,d) 1.0%, (e,f) 1.5%, (g,h) 2.0%.

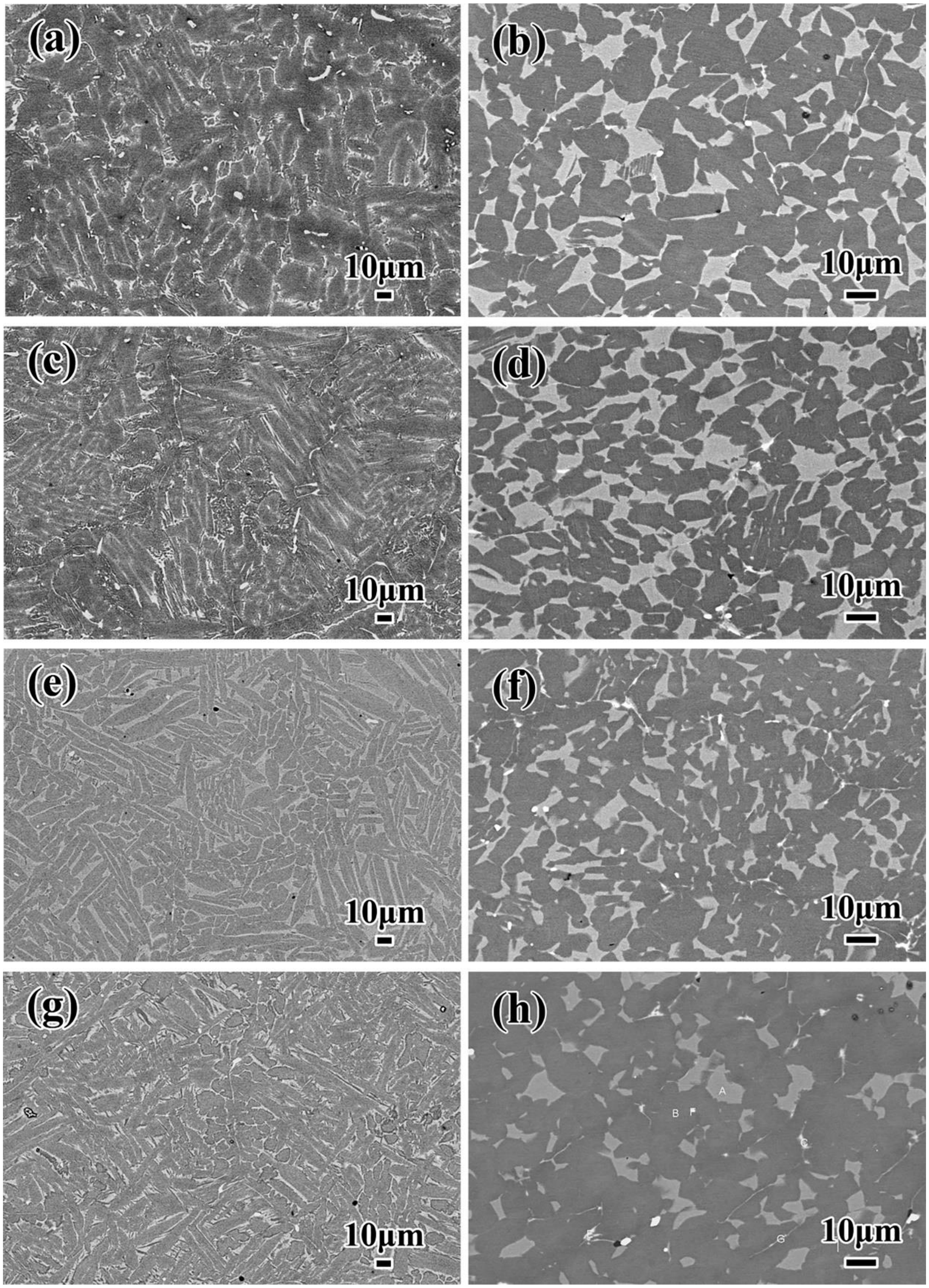

Supplementary Figure S4. BSE SEM images of the as-cast (a,c,e,g) and as-deformed (b,d,f,h) microstructures for alloys with V additions of: (a,b) 0.5%, (c,d) 1.0%, (e,f) 1.5%, (g,h) 2.0%.

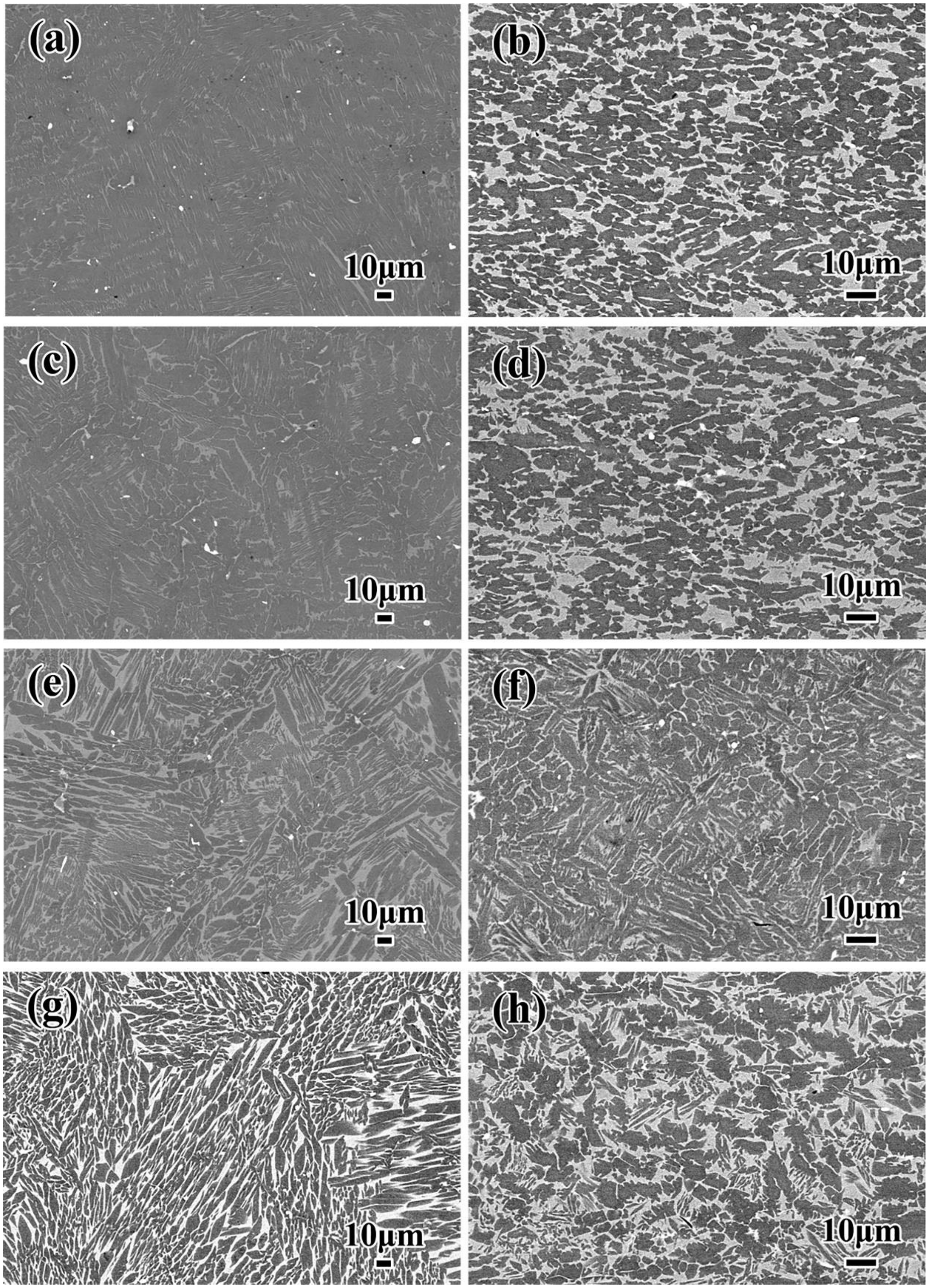

Supplementary Figure S5. BSE SEM images of the as-cast (a,c,e,g) and as-deformed (b,d,f,h) microstructures for alloys with 2.0% Cr plus Mn additions of: (a,b) 0.5%, (c,d) 1.0%, (e,f) 1.5%, (g,h) 2.0%.

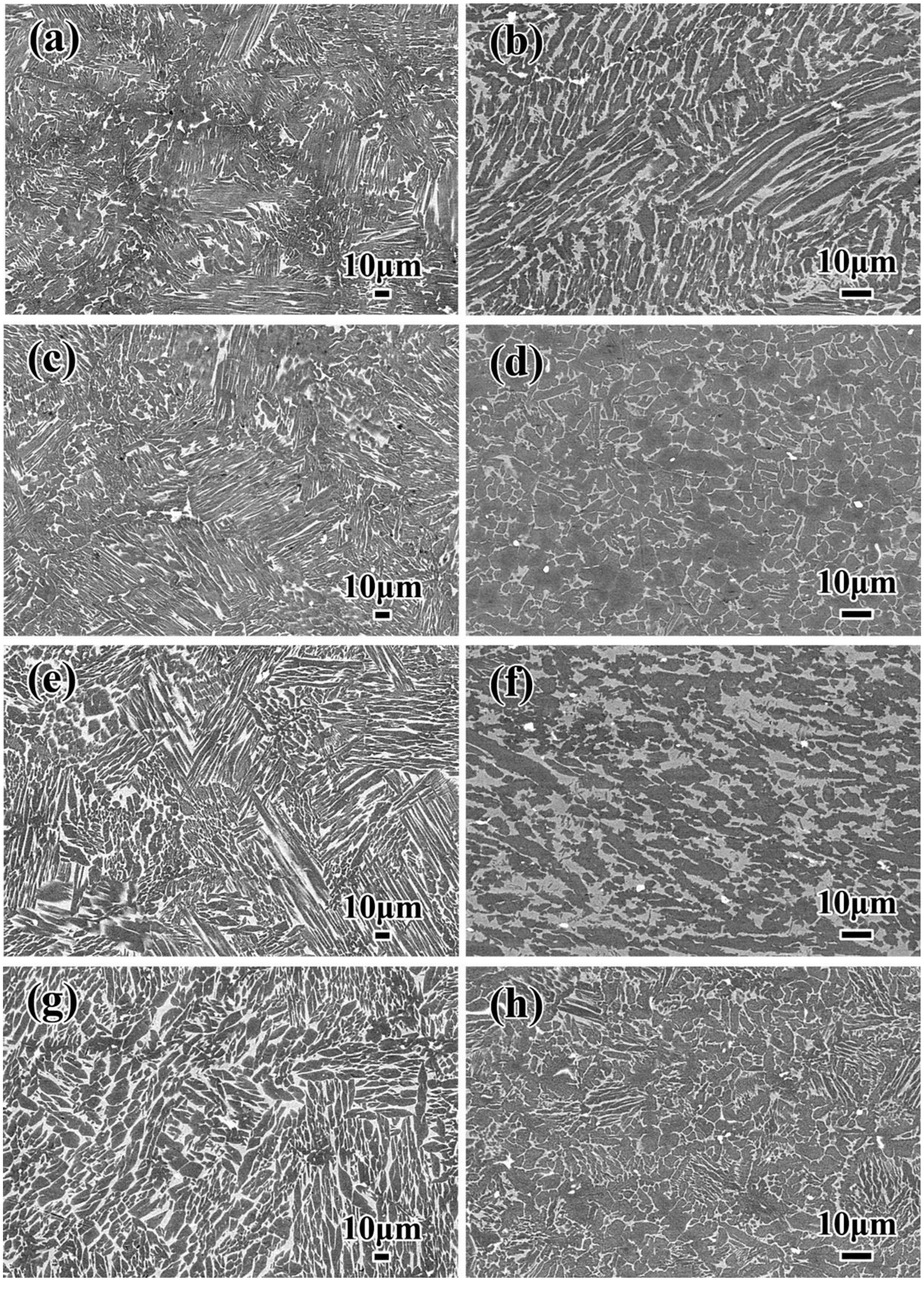

Supplementary Figure S6. BSE SEM images of the as-cast (a,c,e,g) and as-deformed (b,d,f,h) microstructures for alloys with 1.5% Mn plus Cr additions of: (a,b) 0.5%, (c,d) 1.0%, (e,f) 1.5%, (g,h) 3.0%.

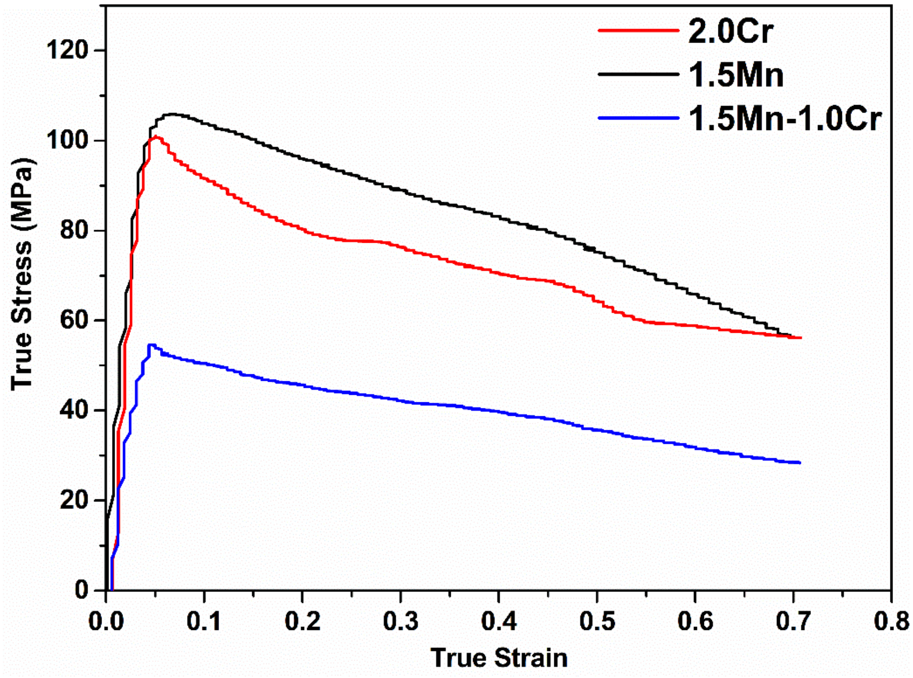

Supplementary Figure S7. True stress-strain curves obtained at $\dot{\varepsilon}$ = 0.05 s-1 from the "optimized" alloy containing 1.5 % Mn + 1.0%Cr, and from the alloys containing 2.0% Cr and 1.5% Mn alone.
